# Case Report: Three's a crowd: a case report examining the diagnostic and pharmacokinetic challenges in HIV-tuberculous meningitis-malaria co-infection

**DOI:** 10.12688/wellcomeopenres.14726.2

**Published:** 2019-01-15

**Authors:** Jayne Ellis, Prosperity C. Eneh, Kenneth Ssebambulidde, Morris K. Rutakingirwa, Mohammed Lamorde, Joshua Rhein, Fiona V. Cresswell, David R. Boulware, Melanie R. Nicol

**Affiliations:** 1 Infectious Diseases Institute, College of Health Sciences, Makerere University, Kampala, Uganda; 2 Hospital for Tropical Diseases, University College London Hospitals NHS Foundation Trust, London, UK; 3 Department of Experimental and Clinical Pharmacology, College of Pharmacy, University of Minnesota, Minneapolis, Minnesota, USA; 4 Division of Infectious Diseases and International Medicine, Department of Medicine, University of Minnesota, Minneapolis, USA; 5 Clinical Research Department, London School of Hygiene and Tropical Medicine, London, UK; 6 LSHTM-MRC-UVRI Uganda Research Unit, Entebbe, Uganda

**Keywords:** tuberculous meningitis, tuberculosis; malaria, HIV/AIDS, pharmacokinetics, drug-drug interactions, case report.

## Abstract

In 2016, 10.4 million cases of tuberculosis (TB) were reported globally. Malaria also continues to be a global public health threat. Due to marked epidemiological overlap in the global burden of TB and malaria, co-infection does occur.

An HIV-infected, 32-year-old male presented with a two-week history of headache with fevers to Mulago National Referral Hospital, Uganda. Five months prior, he was diagnosed with pulmonary TB. He endorsed poor adherence to anti-tuberculous medications. *Mycobacterium tuberculosis* in CSF was confirmed on Xpert MTB/RIF Ultra. On day 2, he was initiated on dexamethasone at 0.4mg/kg/day and induction TB-medications were re-commenced (rifampicin, isoniazid, ethambutol, pyrazinamide) for TBM. He continued to spike high-grade fevers, a peripheral blood smear showed *P. falciparum* parasites despite a negative malaria rapid diagnostic test (RDT). He received three doses of IV artesunate and then completed 3 days of oral artemether/lumefantrine. To our knowledge this is the first published case of HIV-TBM-malaria co-infection.

TBM/malaria co-infection poses a number of management challenges. Due to potential overlap in symptoms between TBM and malaria, it is important to remain vigilant for co-infection. Access to accurate parasitological diagnostics is essential, as RDT use continues to expand, it is essential that clinicians are aware of the potential for false negative results. Anti-malarial therapeutic options are limited due to important drug-drug interactions (DDIs). Rifampicin is a potent enzyme inducer of several hepatic cytochrome P450 enzymes, this induction results in reduced plasma concentrations of several anti-malarial medications. Despite recognition of potential DDIs between rifampicin and artemisinin compounds, and rifampicin and quinine, no treatment guidelines currently exist for managing patients with co-infection.

There is both an urgent need for the development of new anti-malarial drugs which do not interact with rifampicin and for pharmacokinetic studies to guide dose modification of existing anti-malarial drugs to inform clinical practice guidelines.

## Introduction

In 2016, 10.4 million cases of tuberculosis (TB) were reported globally ^1^. Tuberculous meningitis (TBM) accounts for 1–5% of these ^2^. Although TBM can occur in immunocompetent persons, the disease disproportionately affects persons living with HIV and children. Malaria also continues to be a global public health threat. In 2016, an estimated 216 million cases occurred, with 90% of those in Africa ^3^. Due to marked epidemiological overlap in the global burden of TB and malaria, co-infection does occur. In an Angolan retrospective study of 1,906 TB inpatients (37% HIV-infected), *Plasmodium falciparum* co-infection occurred in 38% during hospitalization ^4^. 

TBM/malaria co-infection poses a number of management challenges. Rifampicin, the cornerstone for drug-sensitive TB treatment, is a potent enzyme inducer that increases the expression of several hepatic cytochrome P450 (CYP450) enzymes, including CYP2A6, CYP2B6, CYP2C, and CYP3A isoenzymes, as well as the efflux drug transporter P-glycoprotein ^5^. Peak enzyme induction due to rifampicin is mounted ~ 2 weeks post rifampicin initiation. This induction alters the pharmacokinetics of drugs metabolized by these pathways, reducing plasma concentrations of several anti-malarial medications, including artemisinin-based drugs, quinine and atovaquone/proguanil. In HIV/TBM/malaria co-infection, there are additional interactions between anti-retroviral therapy (ART) and anti-malarial medications ^6^.

Here; we present the case of a hospitalized HIV-infected adult with HIV/TBM/malaria co-infection, highlighting important diagnostic and pharmacokinetic challenges.

## Case report

**Figure 1.**
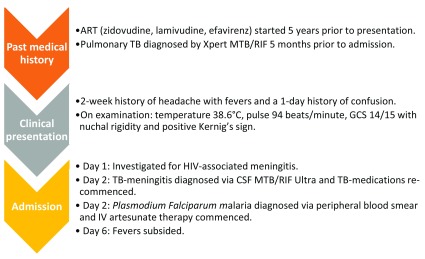
Clinical presentation timeline.

An HIV-infected 32-year-old male presented to Mulago National Referral Hospital, Uganda with a 2-week history of headache with fevers and a 1-day history of confusion ( Figure 1). He had been on ART (zidovudine, lamivudine, efavirenz) and co-trimoxazole prophylaxis for 5 years. 5 months prior, he was diagnosed with pulmonary TB by positive sputum Xpert MTB/RIF (Cepheid, Sunnyvale, CA, USA). He had completed 2 months of induction TB therapy (rifampicin, isoniazid, ethambutol, pyrazinamide) and was 3 months into continuation phase (rifampicin, isoniazid). He endorsed poor adherence to both ART and anti-tuberculous medications.

On examination, the patient was febrile (38.6°C). His blood pressure was 112/71 mmHg, pulse 94 beats/minute, respiratory rate 48, and oxygen saturation 98%. He was wasted, dehydrated, and had overt rigors. His Glasgow Coma Scale was 14/15 with nuchal rigidity and positive Kernig’s sign. Cranial nerves were intact. He had normal tone and power in all limbs. A clinical diagnosis of HIV-associated meningitis was suspected and he was recruited into the ‘Improving Diagnostics and Neurocognitive Outcomes in HIV/AIDS-related Meningitis’ study (registration: ISRCTN42218549). Whilst awaiting further investigations, he received empiric therapy of ceftriaxone 2 g twice daily for possible bacterial meningitis.

A finger stick cryptococcal antigen lateral flow assay (CrAg LFA) (IMMY, Norman, Oklahoma, USA) was negative. Liver and renal function tests were normal. Cerebrospinal fluid (CSF) opening pressure was elevated to 33 cm CSF (normal <20 cm CSF), CSF white cells 590 /µl, protein 419 mg/dl (normal range 15–45 mg/dl), CSF lactate 9.5 mmol/L (normal range <2.5 mmol/l). CSF glucose was unavailable. *Mycobacterium tuberculosis* in CSF was confirmed on Xpert MTB/RIF Ultra; there was no evidence of rifampicin resistance. On day 2, he was initiated on dexamethasone at 0.4 mg/kg/day and induction TB-medications were re-commenced (rifampicin, isoniazid, ethambutol, pyrazinamide) for TBM. The IV ceftriaxone was stopped, his ART was continued. He continued to spike high-grade fevers (39.6°C.) with tachycardia (pulse 118 beats/min). A peripheral blood smear showed *P. falciparum* parasites (1+ trophozoites), despite a negative malaria histidine rich protein-2 (PfHPR2)-based rapid diagnostic test (Malaria *Plasmodium falciparum* Rapid Test Cassette, Vaxpert, Florida, USA). Given his ongoing neurological symptoms, which could be compatible with cerebral malaria, the decision was made to treat for severe malaria. Drug-drug interactions (DDIs) between rifampicin and artemisinin compounds, and rifampicin and quinine are recognized ( Table 1); a decision was made to treat with IV artesunate as the most efficacious anti-malarial for severe malaria ^6^. He received three doses of IV artesunate (3 mg/kg), after which a repeat peripheral blood smear showed no malaria parasites. He then completed 3 days of oral artemether/lumefantrine. His fevers subsided on day 6. He was discharged on day 8; medication adherence counselling was provided for the patient and his guardian and outpatient follow-up was arranged for the following week.

**Table 1.** Summary table of anti-malarial drug interactions with anti-tuberculous and anti-retroviral therapy.

Anti-malarial agents for treatment & prophylaxis	Clinical indication (WHO Malaria guidelines) ^6^	Recommended dose and duration ^6, 10^	Drug metabolism/excretion in healthy adults ^10^	Potential interactions and modification with concurrent rifampicin ^5, 6, 9, 11^	Potential Interactions and modification with concurrent ARVs ^6, 10, 12^
Amodiaquine	1. Uncomplicated *P. falciparum* malaria in combination with artesunate. 2. In combination with sulfadoxine-pyrimethamine for seasonal malaria chemo-prevention in young children	10 (7.5–15) mg/kg/day once a day for 3 days.	Rapidly converted via CYP2C8, to active metabolite desethylamodiaquine. t _1/2_ of amodiaquine ranges from 3–12 hrs, while the active metabolite can vary from 6–18 days.	No significant drug interaction data.	EFV,NVP,AZT/ZVD: ↑plasma conc. &↑LFTS; ↓AUC & risk of hepatotoxicity; increased risk of neutropenia respectively. Avoid use. PIs: Potential ↑plasma conc. Monitor use closely.
Artemether/ lumefantrine	1. Uncomplicated *P. falciparum* malaria as a fixed dose combination with lumefantrine. 2. Intramuscular artemether as an alternative for severe malaria.	Oral Artemeter: 5–24 mg/kg + Oral lumefantrine 29–144 mg/kg twice daily for 3 days. Intramuscular artemether: 3.2 mg/kg.	Converted via CYP3A4/5 (primary), CYP2B6, CYP2C9 and CYP2C19 to active metabolite dihydroartemisinin (DHA). t _1/2_ for artemether and DHA ~1–2 hrs, but up to 7 hrs. Lumefantrine also undergoes metabolism via CYP3A4 to active metabolite desbutyl-lumefantrine. t _1/2_ ~3 days.	Concurrent use results in decreased exposure to artemether/lumefantrine and potential loss of efficacy. ↓ Artemether, DHA, and lumefantrine AUC by 89%, 85%, and 68% respectively. Avoid concurrent use.	EFV, NVP: ↓plasma conc. for both components. Avoid concurrent use. PIs: Potential ↑AUC for lumefantrine. Monitor use closely.
Artesunate	1. Uncomplicated *P. falciparum* malaria in combination with amodiaquine or mefloquine or sulfadoxine/ pyrimethamine. 2. Parental artesunate for severe malaria.	Oral: 4 (2–10) mg/kg/day once a day for 3 days. Parenteral: 2.4 mg/kg unless <25kg/5 years then 3 mg/kg.	Hydrolyzed rapidly to DHA by gut esterases with some contribution from CYP2B6. Very little excreted unchanged in the urine, and inactive metabolites are excreted in the bile. t _1/2_ of artesunate and DHA is <2 hrs.	No published drug interaction data but significant reduction in DHA (the active metabolite) due to CYP450 metabolism may result in reduced efficacy. Monitor closely if concurrent use.	NVP, PIs: Potential ↑ AUC. Monitor if co-administered.
Atovaquone/ Proguanil	1. Prophylaxis of malaria. 2. Uncomplicated malaria in non-endemic regions when primary regimens are not available.	Prophylaxis: Atovaquone 250 mg/ proguanil 100 mg once daily 1 day prior to entering endemic area, during stay, and for 7 days after return. Treatment: Atovaquone 1000 mg/proguanil 400 mg once daily for 3 days.	Converted by CYP2C19 to cycloguanil and 4-chlorophenylbiguanide. Atovaquone is mostly excreted unchanged via fecal route, while proguanil can have renal excretion up to 60%. Atovaquone t _1/2_: adults ~2 to 3 days, pediatric ~1 to 2 days. Proguanil t _1/2_ ~12 to 22 hrs.	Concurrent use may result in reduced atovaquone plasma levels and increase rifampin plasma levels Atovaquone plasma conc.↓ 50% Avoid concurrent use.	EFV, NVP: ↓ conc. of atovaquone, proguanil and possibly cycloguanil. Avoid concurrent use. PIs: Potential ↓ atovaquone AUC. Monitor use closely.
Chloroquine	1. Uncomplicated malaria due to *P.vivax, P. malariae,* *P. ovale and P. knowlesi.*	Initial dose of 10 mg/kg, followed by 10 mg/kg on the second day and 5 mg/kg on the third day.	Metabolized by CYP2C8 and CYP3A4 to active metabolite monodesethychloroquine. Slow elimination via the kidney for both chloroquine and its active metabolite with t _1/2_ ~ 4–12 days.	↓ Chloroquine level might be expected. Monitor closely if concurrent use.	EFV: Potential for QT prolongation. Monitor if co-administered.
Clindamycin	1. Severe or uncomplicated malaria in combination with artesunate or quinine. 2. Preferred: Uncomplicated *P. falciparum* malaria in combination with quinine for first trimester of pregnancy.	10 mg/kg twice daily for 7 days.	Extensive metabolism via CYP3A4 and intestines to N-demethyl and sulfoxide metabolites. Excretion of active drug is via urine (10%) and feces (4%). t _1/2_ is ~ 2–3.5 days.	No significant drug interaction data.	No significant drug interaction data.
Dihydroartemisinin/ piperaquine	1. Uncomplicated *P. falciparum or P. vivax* malaria. 2. Follow up oral treatment in severe malaria.	4 (2–10) mg/kg dihydroartemisinin and 18 (16–27) mg/kg piperaquine once daily for 3 days if weighing ≥ 25 kg. If under 25 kg, then 4 (2.5–10) mg/kg dihydroartemisinin and 24 (20–32) mg/kg piperaquine once daily for 3 days.	Piperaquine induces CYP2E1, inhibits CYP3A4 and CYP2C19 and is a substrate of CYP3A4 (primary), CYP2C9, and CYP2C19. Dihydroartemisinin is an inhibitor of CYP1A2 and substrate of UGT1A9 and UGT2B7. t _1/2_ of dihydroartemisinin ~1 hr, while piperaquine ~13–28 days.	Concurrent use may result in decreased exposure of piperaquine. *In vitro* metabolism by CYP3A4 increased for piperaquine. Consider alternatives and monitor closely if concurrent use.	EFV, NVP: ↓plasma conc. for both components. Potential for QT prolongation with EFV. Monitor use closely. PIs: ↓piperaquine exposure. Monitor if co-administered.
Doxycycline	1. Prophylaxis of malaria. 2. In combination with quinine or artesunate as follow up oral treatment in severe malaria. 3. Uncomplicated falciparum malaria in combination with quinine/ artesunate.	Prophylaxis: 100 mg daily beginning 1 day prior to travel, during travel, and for 4 weeks after return. Treatment: Doxycycline 100 mg twice daily for 7 days	50% hepatic metabolism with extensive enterohepatic cycling Eliminated renally (35–45%) and high conc. of the active drug are also excreted in feces and urine. t _1/2_ ~15–24 hrs.	Concurrent use may result in reduced doxycycline serum conc. and potential loss of doxycycline efficacy. Doxycycline AUC ↓ by 40%. Consider alternatives and monitor closely if concurrent use	No significant drug interaction data.
Mefloquine	1. Prophylaxis of malaria. 2. Uncomplicated *P. falciparum* malaria in combination with artesunate.	Prophylaxis: 250 mg once weekly beginning 1 week prior to travel, continuing weekly and for 4 weeks after return. Treatment: 8.3 (5–11) mg/kg once daily for 3 days.	Metabolized via CYP3A4 to largely inactive metabolites. Undergoes enterohepatic cycling. Minimal renal excretion and excretion is primarily via the bile and feces. t _1/2_ ~13 to 30 days.	Concurrent use results in decreased mefloquine exposure and potential loss of efficacy. Mefloquine AUC ↓ 68%. Avoid concurrent use.	EFV: Potential for QT prolongation. Monitor if co-administered.
Primaquine	1. As an alternative for primary prophylaxis of all malaria. 2. Radical cure of *P. vivax* or *P. ovale* malaria. 3. In combination with ACT or chloroquine for presumptive anti-relapse therapy for extensive exposure to *P. vivax/P. ovale.*	14 day course for relapse prevention and radical treatment: 0.25 mg/kg. Increase to 0.5 mg/kg for frequently relapsing and for primary prophylaxis. Maximum of 30 mg/day. Single dose as an anti- gametocyte medication: 0.25 mg/kg.	Transformed via CYP2C19, CYP2D6 and CYP3A4 to active metabolite 8-(3-carboxyl-1-methylpropylamino)- 6-methoxyquinoline and inactive metabolite carboxyprimaquine. Excretion mainly via the bile and feces but can be excreted unchanged in urine. t _1/2_ ~ 4–7 hrs with longer half-life (15–16 hrs) for the inactive metabolite.	No significant drug interaction data.	No significant drug interaction data.
Quinine	1. Preferred: Uncomplicated *P. falciparum* malaria in combination with clindamycin for first trimester of pregnancy. 2. Parental quinine for severe malaria.	Oral: 650 mg (or two 324-mg capsules (648 mg)) 3 times daily for 3 or 7 days. Parental: Loading dose of 20 mg salt/kg followed by maintenance dose of 10 mg salt/kg every 8 hrs.	Metabolism via CYP3A4 (primary), CYP2C9, CYP1A2 and CYP2D6 to several metabolites including active metabolite 3-hydroxyquinine. Excretion via the kidney (20% unchanged) but potentially small amounts via the bile and saliva. t _1/2_ ~ 10 to 20 hrs.	Concurrent use is associated with a significant decrease in quinine exposure and 5-fold recrudescence rate. Quinine AUC ↓ 75% to 85%. Avoid concurrent use.	EFV, RPV, PIs: Potential for QT prolongation. Monitor if co-administered. NVP: ↓plasma conc. Avoid concurrent use.
Sulfadoxine/ pyrimethamine	1. Intermittent prophylaxis in pregnant women in 1 ^st^/2 ^nd^ pregnancy and infants in areas of mid- high intensity malaria transmission. 2. In combination with atovaquone for seasonal malaria chemoprevention in young children in areas of high intensity malaria transmission.	Single administration of at least 25/1.25 (25–70 / 1.25–3.5) mg/kg sulfadoxine /pyrimethamine given as a single dose on day 1.	Metabolism via acetylation (60%), glucuronidation (10%) and conjugation. There is about 30% renal excretion of unchanged drug for both sulfadoxine and pyrimethamine. t _1/2_ ~9.5 days and for pyrimethamine ~2.5 –6 days.	No significant drug interaction data.	No significant drug interaction data.

t _1/2_, elimination half-life; EFV, efavirenz; NVP, nevirapine; AZT/ZVD, zidovudine; DHA, dihydroartemisinin; PI, protein inhibitors; AUC, area under the curve; ACT, artemisinin-based combination therapy; RPV, rilpivirine.

## Discussion

This case demonstrates the diagnostic and treatment challenges encountered when managing patients with advanced HIV and intercurrent infections. Protracted high-grade fevers are not an uncommon feature of TBM, even after appropriate anti-tuberculous treatment has been commenced, and it is important to remain vigilant for co-infections. Co-infection with HIV-TB-malaria is well recognized ^4^; however, to our knowledge this is the first published case of HIV-TBM-malaria co-infection.

Due to the potential overlap in symptoms and signs between TBM and malaria (fever, confusion, reduced level of consciousness, seizures, sepsis), access to accurate parasitological diagnostics is essential. Light microscopy (Giemsa stain) remains the gold standard parasitological diagnostic, but the World Health Organization (WHO) recommends immunochromatographic rapid diagnostic testing (RDT) in settings with limited laboratory facilities ^6^. RDTs—which detect *Plasmodium* antigens such as PfHPR2, *Plasmodium* lactate dehydrogenase (pLDH) or plasmodial aldolase—are the backbone of expanding access to malaria diagnostics in resource-limited settings. Since their introduction in the late 1990s, the number of RDTs available, and the scale of their use, has increased rapidly. A meta-analysis of 74 studies assessing accuracy of PfHRP-2 RDTs for diagnosis of uncomplicated *P. falciparum* malaria in endemic settings reported average sensitivity and specificity (95% CI) of 95.0% (93.5-96.2%) and 95.2% (93.4-99.4%), respectively ^7^. However RDTs do have several limitations: poor sensitivity at low parasite densities; susceptibility to the prozone effect (PfHRP2-detecting RDTs); false-negative results due to PfHRP2 gene deletions; false-positive results caused by other infections, and susceptibility to heat and humidity ^8^. The largest problem historically has been poor manufacturing quality, which the World Health Organization (WHO) malaria product testing programme has addressed. However, Vaxpert does not participate in this WHO quality assurance program. As demonstrated by this case, false negatives can occur.

The WHO recommends treatment with an artemisinin derivative for both uncomplicated (oral artemisinin combination therapy (ACT) for 3 days) and complicated falciparum malaria infections (intravenous or intramuscular artesunate for at least 24 hours and until the patient can tolerate oral medications, at which stage 3 days of ACT should be completed) ^6^. It is recommended that a lipid rich meal is eaten prior to taking lumefantrine to optimise absorption. Despite the recognition of potential DDIs between first-line anti-malarial drugs and rifampicin, no treatment guidelines currently exist for managing patients with TB/malaria co-infection. The WHO states that there is currently a lack of evidence to recommend dosage modifications but advises clinicians of increased risk of recrudescent infections due to DDIs in co-infected patients ^6^.

In a DDI study in HIV-infected Ugandans to investigate the pharmacokinetics of artemether, dihydroartemisinin (DHA) and lumefantrine during rifampicin intake, co-administration with rifampicin resulted in a significantly lower exposure (area under the curve between 0 and 12 hours post-dosing) to artemether (89% lower) and DHA (85% lower) ^9^. Co-administration of artemether-lumefantrine and rifampicin should therefore be avoided. There are no published studies examining concomitant administration of IV artesunate and rifampicin. However, as DHA (the active metabolite of artesunate) is metabolized by CYP450 enzymes ( Table 1), there is a theoretical risk that co-administration with rifampicin would result in reduced plasma DHA concentrations and a reduction in efficacy ^5^. Oral agents may undergo metabolism in the gut and the liver prior to reaching the systemic circulation, but intravenous drugs are directly administered to the systemic circulation and so reductions in DHA exposure with intravenous artesunate could be potentially of lower magnitude than what was seen with oral artemether.

Similarly, the bioavailability of quinine—metabolized almost exclusively via CYP450 (CYP3A4 and CYP2C19) enzymes—is significantly reduced when co-administered with rifampicin and has been associated with a clinically significant reduction in efficacy. Adults treated for uncomplicated falciparum malaria were randomized to receive oral quinine either alone or in combination with rifampin; recrudescence rates were five times higher (15/23; 65%) in the rifampicin arm than those treated with quinine alone (3/25; 12%, *P*<0.001) ^11^. It is recommended that for patients already receiving rifampicin, quinine doses should be increased. However, to date, no guidance on dose-adjustment strategies have been published, and although it is advised that therapeutic drug monitoring may be useful, this is not feasible in most parts of the world where co-infection occurs ^5^.

A range of anti-malarial drugs used in the treatment of non-severe malaria and for prophylaxis also have documented DDIs with rifampicin including: atovaquone; chloroquine; piperaquine; mefloquine and doxycycline ( Table 1). As no clinical guidelines currently exist regarding dose modification, these drugs should be used with caution in patients concurrently receiving rifampicin.

Many antiretrovirals also interact with the CYP450 system ( Table 1). For example, co-administration with efavirenz reduces exposure to the active components of artemether and lumefantrine 46% and 21%, respectively. However, the net induction effects of the use of both rifampicin and efavirenz on antimalarial compounds has not been quantified. Similarly, protease inhibitors are potent CYP450 inhibitors and their combined effect with rifampicin induction is unknown.

This case highlighted the difficulty in managing co-infected patients and the lack of informed treatment options. New anti-malarial medication development have the potential to offer improved potency, tolerability, and shorter treatment duration but drug- drug interactions are likely to persist. In cases of suspected significant interactions, healthy volunteer studies may play a role in informing management as efficient study designs allow for small sample sizes. Our group has also employed building in pharmacokinetic sampling and analysis into existing clinical trials. Coupling sparse or opportunistic sampling with pharmacokinetic modeling and simulation can allow for increased precision in dose selection.

## Conclusions

In conclusion, patients with HIV-TBM-malaria co-infection present a number of management challenges. The potential symptom overlap in clinical presentation means that clinicians must remain vigilant for co-infection and access to reliable parasitological diagnostics is imperative. As malaria RDT use continues to expand, it is essential that clinicians are aware of the potential for false negative results. Therapeutic options for TB-malaria co-infection are limited due to DDIs. There is both an urgent need for the development of new anti-malarial drugs which do not interact with rifampicin and for pharmacokinetic studies to guide dose modification of existing anti-malarial drugs to inform clinical practice guidelines.

## Data availability

*All data underlying the results are available as part of the article and no additional source data are required.*

## Consent

The Improving Diagnostics and Neurocognitive Outcomes in HIV/AIDS-related Meningitis study protocol was approved by Ugandan and Minnesota IRBs. Written informed consent from the patient’s next of kin was given for publication of that patient’s clinical details as the patient himself lacked the mental capacity to consent.
