# Supplementary material for: Case Report: Three's a crowd: a case report examining the diagnostic and pharmacokinetic challenges in HIV-tuberculous meningitis-malaria co-infection
Source: Wellcome Open Res. 2019 Jan 15;3:111. Originally published 2018 Sep 6. [Version 2] doi: 10.12688/wellcomeopenres.14726.2 (PMC6343228; doi:10.12688/wellcomeopenres.14726.2)
Supplement: Peer review history

# Peer review: Referee response for version 2

**DOI:** 10.21956/wellcomeopenres.16387.r34594

**Published:** 2019-01-22

**Authors:** Catriona Waitt

**Affiliations:** 1 Department of Molecular and Clinical Pharmacology, University of Liverpool, Liverpool, UK; 2 Infectious Diseases Institute, Makerere University College of Health Sciences, Kampala, Uganda

Thank you for addressing the comments I raised in the first review. I am happy that these have been addressed and have no further comments.

I have read this submission. I believe that I have an appropriate level of expertise to confirm that it is of an acceptable scientific standard.

# Peer review: Referee response for version 2

**DOI:** 10.21956/wellcomeopenres.16387.r34593

**Published:** 2019-01-16

**Authors:** Tom Boyles

**Affiliations:** 1 Wits Reproductive Health and HIV Institute, University of the Witwatersrand, Johannesburg, South Africa

The authors have addressed the minor issues I raised in my review and I now think the case report is ready for indexing.

I have read this submission. I believe that I have an appropriate level of expertise to confirm that it is of an acceptable scientific standard.

# Peer review: Referee response for version 1

**DOI:** 10.21956/wellcomeopenres.16043.r34324

**Published:** 2018-12-07

**Authors:** Catriona Waitt

**Affiliations:** 1 Department of Molecular and Clinical Pharmacology, University of Liverpool, Liverpool, UK; 2 Infectious Diseases Institute, Makerere University College of Health Sciences, Kampala, Uganda

Thank you very much for this report. Whilst this may be the first published report of the three infections occurring concurrently, it is likely that in practice these comorbidities and resulting drug-drug interactions are much more common, and hence it is an important contribution to the literature. Both the diagnostic challenges in this case, and the limitations of the best available therapeutic options given the drug-drug interactions are clearly presented.

 The table summarising the drug interactions is very helpful.

 For those who may be less familiar with managing such patients, was the case unusual? Is it common to present with TB meningitis five months into therapy where there has been apparent adherence?

 Was the ceftriaxone discontinued once the positive TB result from CSF was obtained? (I presume so, but it is not explicitly stated)

 I would be interested to know about any follow-up post discharge. Was anything done differently, for example support for adherence etc?

 The conclusion finishes with reiterating the ‘urgent need for the development of new anti-malarial drugs which do not interact with rifampicin and for pharmacokinetic studies to guide dose modification of existing anti-malarial drugs to inform clinical practice guidelines’ – I entirely agree with this statement, but given the challenges and diversity in the population in question which has been described, I would also have liked a couple of sentences in the discussion  about how such studies could best be designed. What would be ideal? Logistically, how could such a population be drawn together to provide meaningful data? Do healthy volunteer studies have a part to play here? Are the authors involved in any existing work to try and address some of these gaps? Are they aware of any other work that is ongoing?

 Minor points

 CSF opening pressure should be measured in cm CSF, not cm H20.

I have read this submission. I believe that I have an appropriate level of expertise to confirm that it is of an acceptable scientific standard, however I have significant reservations, as outlined above.

# Peer review: Referee response for version 1

**DOI:** 10.21956/wellcomeopenres.16043.r33877

**Published:** 2018-09-17

**Authors:** Tom Boyles

**Affiliations:** 1 Wits Reproductive Health and HIV Institute, University of the Witwatersrand, Johannesburg, South Africa

This is a well written case report highlighting an important issue for clinicians practicing in areas where malaria, HIV and TB are endemic.

 Prompted by a case they saw, the authors helpfully summarise the potential DDIs between anti-malarial medications and anti-tuberculous and anti-retroviral medications in common use. This issue deserves publication in its current form with consideration of a few minor issues.

 I have only minor comments to make.

 -Was the ceftiaxone stopped as soon as the Xpert was positive? This would be appropriate given the low pre-test probability of bacterial meningitis (2 week history) and the identification of a an alternative definite diagnosis. It might be helpful to remind readers that 'finishing the course' of an antibiotic in this situation is not necessary and can cause harm.

 -It would be helpful to confirm that the Xpert MTB/RIF Ultra confirmed rifampicin sensitivity as the patient was clearly at risk of DR-TB.

 -It would be worth pointing out that it takes around 2 weeks for the full effects of enzyme induction by rifampicin to be felt. If malaria is diagnosed at a similar time to TB, as in this case, the initial effects of enzyme induction might be limited, but increase over time which would increase the challenge of finding the appropriate dose.

 - It is never a bad thing to remind people of the need for a lipid meal before taking lumafantrine in order to achieve adequate absorption, something which is often neglected.

I have read this submission. I believe that I have an appropriate level of expertise to confirm that it is of an acceptable scientific standard, however I have significant reservations, as outlined above.
